# Impact of multiple-dose versus single-dose inhaler devices on COPD patients’ persistence with long-acting β_2_-agonists: a dispensing database analysis

**DOI:** 10.1038/npjpcrm.2014.69

**Published:** 2014-10-02

**Authors:** Job FM van Boven, Joost J van Raaij, Ruben van der Galiën, Maarten J Postma, Thys van der Molen, PN Richard Dekhuijzen, Stefan Vegter

**Affiliations:** 1 Unit of PharmacoEpidemiology and PharmacoEconomics, Department of Pharmacy, University of Groningen, Groningen, The Netherlands; 2 Department of Primary Care, University Medical Centre Groningen, University of Groningen, Groningen, The Netherlands; 3 Department of Pulmonary Diseases, Radboud University Medical Centre, Nijmegen, The Netherlands

## Abstract

**Background:**

With a growing availability of different devices and types of medication, additional evidence is required to assist clinicians in prescribing the optimal medication in relation to chronic obstructive pulmonary disease (COPD) patients’ persistence with long-acting β_2_-agonists (LABAs).

**Aims:**

To assess the impact of the type of inhaler device (multiple-dose versus single-dose inhalers) on 1-year persistence and switching patterns with LABAs.

**Methods:**

A retrospective observational cohort study was performed comparing a cohort of patients initiating multiple-dose inhalers and a cohort initiating single-dose inhalers. The study population consisted of long-acting bronchodilator naive COPD patients, initiating inhalation therapy with mono-LABAs (formoterol, indacaterol or salmeterol). Analyses were performed using pharmacy dispensing data from 1994 to 2012, obtained from the IADB.nl database. Study outcomes were 1-year persistence and switching patterns. Results were adjusted for initial prescriber, initial medication, dosing regimen and relevant comorbidities.

**Results:**

In all, 575 patients initiating LABAs were included in the final study cohort. Among them, 475 (83%) initiated a multiple-dose inhaler and 100 (17%) a single-dose inhaler. Further, 269 (47%) initiated formoterol, 9 (2%) indacaterol and 297 (52%) salmeterol. There was no significant difference in persistence between users of multiple-dose or single-dose inhalers (hazard ratio: 0.98, 95% confidence interval: 0.76–1.26, *P*=0.99). Over 80% re-started or switched medication.

**Conclusions:**

There seems no impact of inhaler device (multiple-dose versus single-dose inhalers) on COPD patients’ persistence with LABAs. Over 80% of patients who initially seemed to discontinue LABAs, re-started their initial medication or switched inhalers or medication within 1 year.

## Introduction

Chronic obstructive pulmonary disease (COPD) places a high burden on both public health and health-care budgets of patients and the government.^1,2^ Despite no curative treatment being available, inhaled medication has been shown to reduce disease symptoms.^3,4^ However, effectiveness of current medication has mainly been assessed in controlled clinical trial settings, which may not properly reflect real-life behavior of patients. Furthermore, these trial populations do not fully represent the characteristics of the average COPD population.^5^ Both factors contribute to a real-life adherence to COPD medication that is far beyond adherence reported in clinical trials.^6,7^ Recently, non-adherent patients were highlighted as a target for interventions because of the significant clinical and economic impact of non-adherence on patients with COPD.^8^ Factors associated with non-adherence with COPD medication include age, comorbidity, disease severity and class of medication.^9,10^ However, only a few factors may be modified by the prescribing clinician. Compared to the abundant number of adherence studies in asthma patients, studies on patients with COPD are limited.^6,7,11–13^ In particular, studies assessing the impact of the type of inhaler device on medication adherence in patients with COPD are lacking.^14^ With a growing availability of different types of medication and inhaler devices, additional evidence is required to assist clinicians in prescribing the optimal medication in relation to their patients’ adherence. Adherence does encompass compliance (the extent of acting according to the prescribers’ advice) and persistence (duration of time from initiation till discontinuation of therapy).^15^ The objective of this study is to assess the impact of the type of inhalation device on COPD patients’ persistence with, and switching between, long-acting respiratory medication. The focus on persistence was chosen as this could be objectively measured using a dispensing database, whereas compliance would require face-to-face assessment of patients. In particular, we focused on persistence with long-acting β_2_-agonists (LABAs), where we compared single-dose versus multiple-dose inhalers.

## Materials and Methods

### Data source

Data were obtained from the InterActionDatabase (IADB.nl, available from http://www.iadb.nl). The IADB.nl database covers pharmacy-dispensing data from over 500,000 patients from 55 community pharmacies located in the Northern Netherlands between 1994 and 2012. Dispensing data contain information on drug name, product specifications, anatomical therapeutic chemical code, prescriber, amount of delivered drug units, daily dose and patient characteristics such as date of birth, gender and socioeconomic status, regardless of health-care insurance status of the patients. Thus, the database is representative for the real-world situation in the Netherlands. Medication dispensed in hospitals is not included. The IADB.nl database has been validated for drug utilisation studies^16^ and has been used in previous persistence studies.^17,18^ In the Netherlands, patients usually redeem their prescriptions at the same pharmacy allowing a complete longitudinal follow-up.

### Study population

As the database lacked data on clinical diagnosis, COPD patients were selected based on initiation of inhaled respiratory medication above the age of 55 years, in line with previous studies.^7,12^ Treatment initiation was defined as at least one prescription of LABAs for inhalation (salmeterol (R03AC12), formoterol (R03AC13), or indacaterol (R03AC18)). The group of LABAs was chosen as for this group both dry powder inhalers (DPIs) and pressurised metered dose inhalers are available. Moreover, within the DPI group, both single-dose as well as multiple-dose inhalers are available, making this the group of long-acting bronchodilators where the influence of inhaler device could be most extensively tested. Starters were defined as not having used any long-acting respiratory medications (LABAs, inhaled corticosteroids (ICS) or long-acting muscarinic antagonists) for at least 2 years before treatment initiation. To make sure that a complete overview of history and follow-up was available, patients needed to be registered in the database at least 2 years before and 1 year after initiation of therapy. We only included patients who started one type of inhaled medication at treatment initiation (that is, patients who started a LABA and at the same date ICS or long-acting muscarinic antagonist were excluded). To minimise the possibility to include (late onset) asthma patients, we excluded patients using typical asthma medication (antihistamines (R06), anti-allergics (R03BC), leukotriene receptor antagonists (R03DC), and omaluzimab (R03DX05)) within 1 year after long-acting beta agonist initiation.

### Persistence

Continuous drug use in the first year was calculated by the total number of doses dispensed from therapy initiation, divided by the prescribed daily dose. A gap of maximal 60 days without medication coverage within the period of continuous use was allowed to consider a patient being still persistent to medication, that is, patients who did not have any gaps exceeding 60 days in the 1-year period after therapy initiation was considered persistent. This 60-day gap is a common cutoff and was used in various previous studies assessing persistence with COPD medication.^6,7^

### Switching

Part of the nonpersistent patients were those who did discontinue their initial inhaler or medication but did not completely discontinue the use of any long-acting medication at all (that is, they switched therapy). Therefore, follow-up of non-persistent (to initial therapy) patients was monitored by assessing patients’ medication profiles after the date of discontinuation of their initial therapy. For all patients, follow-up was at least 1 year from the date of treatment initiation; however, to be able to assess switching patterns in the year after the date of treatment discontinuation, follow-up needed to be longer. Therefore, we defined a subcohort where patients needed to have a follow-up of at least 2 years. After discontinuation of initial treatment, we assessed the following possibilities: a switch to a different inhaler within the DPI class (that is, from single- to multiple-dose inhaler or *vice versa*), a switch to a LABA-pressurised metered dose inhaler, a switch to a different bronchodilator class (LABA/ICS or long-acting muscarinic antagonist), a restart with initial type of medication/inhaler or permanent discontinuation of any long-acting bronchodilator.

### Defining the single-dose and multiple-dose cohorts

Two cohorts were defined based on the type of LABA-DPI initiated by the patients. We made a distinction between single-dose and multiple-dose DPI inhalers. Single-dose inhalers require loading of separate capsules before using the device.^19,20^ Examples include the Handihaler, the Cyclohaler, the Rotahaler and the Breezhaler. Multiple-dose inhalers contain a fixed amount of doses (usually between 60 and 120) that can be taken successively until the device runs out of doses. Examples include the Diskus, the Turbuhaler and the Novolizer. We hypothesised that the use of single-dose inhalers required more complex and time-consuming handlings in order to perform proper inhalation and would therefore decrease treatment persistence.

### Covariates

Although this study focused on the medication and inhaler devices, persistence may be altered by several other parameters. The following parameters were compared between the two cohorts: age, gender, socioeconomic status, initial prescriber, comorbidity (described in more detail below), number of short courses of oral corticosteroids, use of short-acting bronchodilators, initial medication and dosing regimen. We performed a multivariate analysis to correct for parameters that significantly differed (*P*<0.05) between the two cohorts.

#### Comorbidity

Included comorbidities were selected based on frequently reported comorbidities from previous studies.^21^ If available, proxies from earlier studies were used to identify comorbidities based on medication use.^22–24^ Diabetes was identified by use of glucose-lowering drugs, that is, drugs with anatomical therapeutic chemical code ‘A10%’. Dyslipidaemia was identified by use of lipid-lowering drugs (C10). Heart failure and ischaemic heart disease were identified by prescription of digoxin or loop diuretics (C01AA05 or C03C) and C01DA, respectively. Patients not using previously mentioned anatomical therapeutic chemical codes but using other cardiovascular drugs (C02, C03, C07, C08 or C09) were classified as patients suffering from other cardiovascular disorders. Other diseases included osteoporosis (M05B), anxiety (N05B and N05C), dementia (N06B), depression (N06A), rheumatic arthritis (M01 and M02) and hypothyroid disease (H03). Patients needed to have at least two of these prescriptions within 1 year after COPD treatment initiation to be considered a chronic user of these comedications.

### Statistics

Parameters of the two cohorts were compared using Fisher’s exact tests or Mann–Whitney *U*-tests, where appropriate.

Kaplan–Meier survival analyses were used to visualise patients’ persistence over a 1-year-time period, comparing patients initiating multiple-dose inhalers and single-dose inhalers. In multivariate Cox proportional hazard analyses, results were adjusted for parameters that differed between the two cohorts with a 95% confidence level (*P*<0.05). Statistics were performed with IBM SPSS Statistics 22 (IBM Corp., Armonk, NY, USA).

### Handling of missing data

Patients without gender or date of birth information were excluded from the cohort, just as patients who died in the year after treatment initiation. In case of missing data on essential parameters such as dosing regimen or amount of inhalers dispensed, patients were also excluded.

### Sensitivity analyses

In order to assess the sensitivity of the gap that was chosen to define persistence, we also performed an analysis with a 30- and a 90-day gap. These analyses will ease generalisability of results by allowing comparison with previous studies using different definitions of persistence. Sensitivity analyses were performed for patients with at least two prescriptions for long-acting bronchodilators (including LABA-ICS or long-acting muscarinic antagonist) in the year after treatment initiation, that is, one-time users (for example, in case of an incident exacerbation) were excluded.

## Results

### Population selection process

In all, 1,838 potential COPD patients initiated a LABA of interest (formoterol, indacaterol or salmeterol) between 1996 and 2011. After exclusion of likely asthma patients (223), 1,615 patients remained. Of these, 595 patients had no complete information records on essential parameters to calculate persistence (for example, dosing regimen or amount of inhalers) and were excluded, leaving 1,020 patients. Among them, 207 started two long-acting inhalers on the same date and were excluded, leaving 813 patients. Of those, 238 (29%) started with a pressurised metered dose inhaler and 575 (71%) started with a DPI. Of all the DPI users, 475 (83%) initiated a multiple-dose inhaler and 100 (17%) a single-dose inhaler.

**Figure 1:**
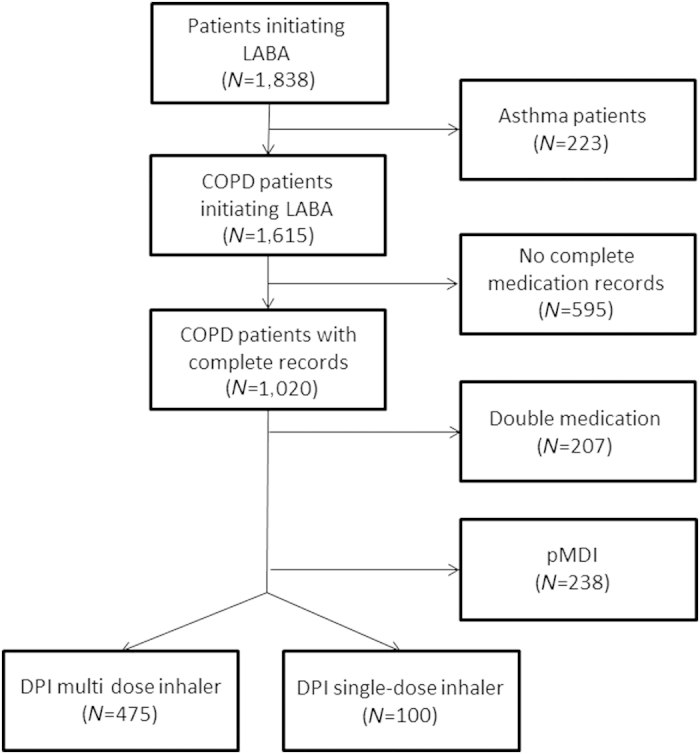
Flow diagram of the population selection process. Asthma patients, excluded based on use of antihistamines, anti-allergics, leukotriene receptor antagonists or omaluzimab; COPD, chronic obstructive pulmonary disease; double medication, initiating of two types of inhaled medication at the same date; DPI, dry powder inhaler; LABA, long-acting β_2_-agonist; pMDI, pressurised metered dose inhaler.

A flow diagram is provided in Figure 1. Patient characteristics, per cohort, are presented in Table 1. Parameters that significantly differed (*P*<0.05) were the initial prescriber, comorbidities (lipid-metabolism disorders and psychosis, in particular), the initial medication and the dosing regimen.

### Analysis of persistence

Overall, 1-year persistence in the basecase scenario (60-day gap, minimal one prescription) was 14.4%. Persistence between users of multiple-dose and single-dose inhalers did not significantly differ (hazard ratio: 0.96, 95% confidence interval: 0.76–1.22, *P*=0.74). In the multivariate analysis, results did not change (hazard ratio: 0.98, 95% confidence interval: 0.76–1.26, *P*=0.99).

### Sensitivity analyses

#### Impact of the gap

In sensitivity analyses, overall 1-year persistence changed when increasing or decreasing the gap allowed for being considered persistent. Decreasing to a 30-day gap reduced overall persistence to 9.6%, whereas increasing to a 90-day gap improved overall treatment persistence to 19%.

#### Excluding one-time users

**Figure 2:**
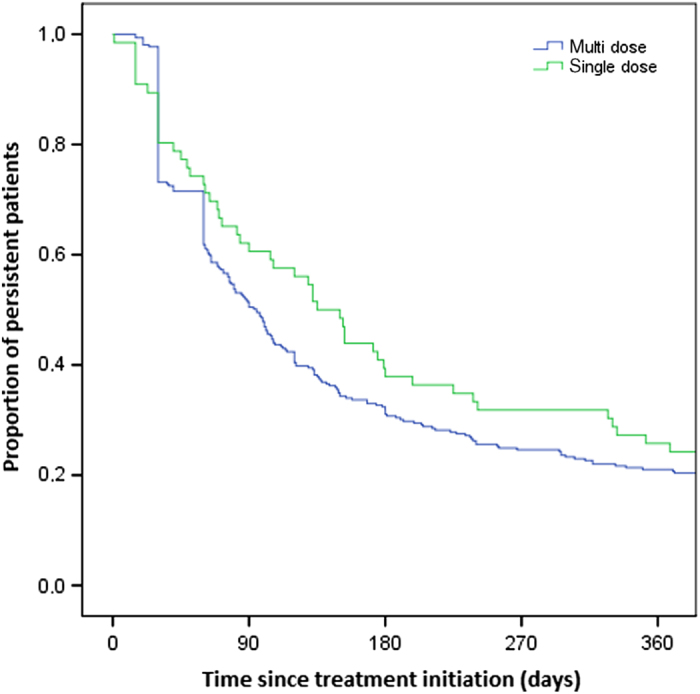
Persistence with initial inhaled beta agonists of patients (*N*=375) with minimal two dispenses for bronchodilators, allowing a maximum gap of 60 days: multiple-dose versus single-dose inhalers.

Including only patients with two prescriptions or more resulted in a cohort of 375 patients initiating LABAs. Sixty-six (18%) patients initiated a single-dose inhaler and three hundred nine (82%) a multiple-dose inhaler. Overall persistence was 21.9%. There was no significant difference between persistence of users of single-dose or multiple-dose inhalers (25% vs. 21%, *P*=0.24). Persistence with initial medication over time is graphically shown in Figure 2. Note that in this figure patients were marked non-persistent when they did not redeem their next prescription of their *initial* inhaler within 60 days after the theoretical end date of their previous prescription (when used as prescribed). Take into account that the dosing regimen varied between one and four times per day. The time point of non-persistence is the theoretical end date of their last prescription *before* their first gap.

#### Allowing switching

**Figure 3:**
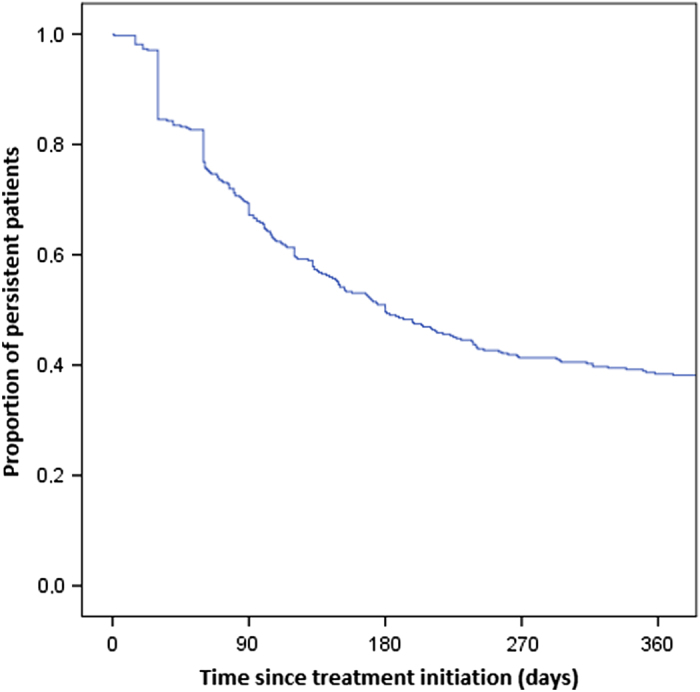
Overall persistence with any long-acting inhaled medication of patients (*N*=375) with minimal two dispenses for bronchodilators, allowing a maximum gap of 60 days and allowing switching.

In Figure 3, persistence with any long-acting medication over time is shown for a cohort that included only patients with two or more prescriptions and allowing switching, resulting in an overall persistence of 38.4%.

#### Switching patterns

**Figure 4:**
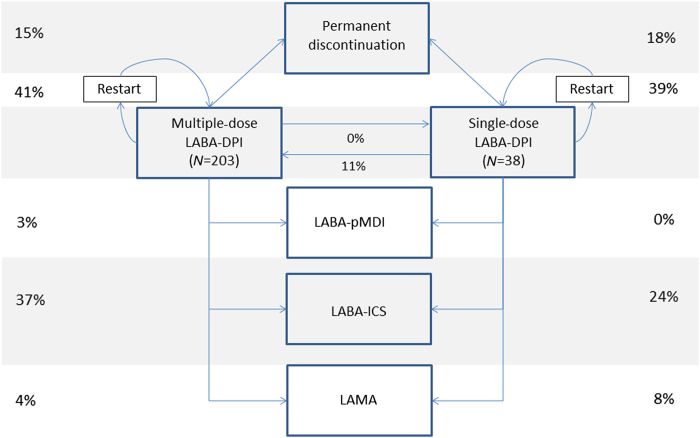
Switching patterns after the first 60-day gap in patients with at least two dispenses and 2 years follow-up: single-dose versus multiple-dose inhalers. LABA, long-acting β_2_-agonist; ICS, inhaled corticosteriod; LAMA, long-acting muscarinic antagonist; DPI, dry-powder inhaler; pMDI, pressurised metered-dose inhaler.

Figure 4 shows the switching patterns of non-persistent patients after their first 60-days gap. Note that the switching analysis was performed in a subcohort (*N*=307) with at least two dispensings for long-acting bronchodilators (that is, one-time users are not included) and a follow-up of at least 2 years from treatment initiation. In this cohort, 38 out of 53 single-dose inhaler users were non-persistent and 203 out of 254 multiple-dose inhaler users. From Figure 4 it is shown that ~40% of all patients (disregarding their inhaler type) re-started their initial therapy within 1 year after their first gap. Less than 19% of the patients with a gap did permanently discontinue any type of long-acting respiratory therapy. A considerable percentage of patients switched to fixed dose inhalers of LABA and ICS. Of the single-dose inhaler users, 11% switched to multiple-dose inhalers of the same medication, whereas none of the multiple-dose inhaler users switched to single-dose inhalers with the same medication.

## Discussion

### Main findings

This study showed no significant impact of inhaler device on COPD patients’ persistence with LABAs: persistence of patients initiating multiple-dose inhalers was comparable to patients initiating single-dose inhalers. Whereas overall persistence with LABAs seemed relatively low at first sight, this study showed that ~40% of all patients (disregarding their inhaler type) re-started their initial therapy within 1 year. Only 15% of the patients who seemed to discontinue based on a 60-day treatment gap did indeed permanently discontinue any type of long-acting respiratory therapy. A considerable percentage of patients (about one-third) switched to fixed dose inhalers of LABA and ICS. Of the single-dose inhaler users, 11% switched to multiple-dose inhalers of the same medication, whereas none of the multiple-dose inhaler users switched to single-dose inhalers with the same medication.

### Interpretation of findings in relation to previously published work

This study is the first describing the relationship between inhaler devices and persistence with COPD maintenance medication. In asthma, DPI devices have been linked to improved adherence compared with pressurised metered dose inhalers.^25^ Specific studies in COPD patients assessing the impact of inhaler device on adherence or persistence are lacking.^14^ In general, medication persistence in our study was far short of optimal, for both multiple-dose as well as single-dose inhalers. This relatively low persistence is in line with findings from previous studies;^6,7^ however, this study showed that over 80% of patients who initially seemed to discontinue LABAs, based on a 60-day treatment gap, re-started their initial medication or switched to different types of inhaler or medication within 1 year after discontinuation. The high percentage of switching may suggest that for some prescribers it takes some time to find the optimal inhaler and medication to suit their patients’ preferences regarding persistent use. However, whereas an optimal inhaler may favour patients’ persistence, improved disease control and better clinical outcomes are not guaranteed. Even though in controlled clinical trial settings comparing inhaler devices no differences in outcomes were observed,^26^ in daily practice, many problems with inhalers have been described.^19,20,27^ Main problems are related to poor inhalation technique,^28^ resulting in suboptimal deposition in the lungs, oropharyngeal side effects^29^ and ultimately poor disease control.^30^ Instruction on inhalation techniques has been shown to not only improve adherence but was also associated with better health-related quality of life.^31^ Apart from inhalation technique and lung deposition issues, one can argue whether improved health outcomes are solely achieved by adherence to respiratory medication or that COPD patients’ (self-management) skills and behaviour determine an important part of improved outcomes. Notably, good adherers with COPD placebo inhalers showed comparable outcomes on mortality and hospitalisation rates as good adherers to real medications.^32^ Vestbo *et al.* ^32^ describe this phenomenon as the ‘healthy adherer effect’. It is suggested that COPD patients who are good adherers to their medication have a healthier lifestyle in general (similar to more awareness of the importance of smoking cessation, a healthy diet and intensity of exercise). In other words, medication non-adherence may be an indicator of patients’ shortage of disease- and self-management skills. This reveals an important opportunity for the health-care provider: actively search for non-adherent COPD patients (for example, by medication dispense records) and target your interventions to these patients. Using this strategy, not only health-care providers’ time is more efficiently spent but also the cost effectiveness of disease management interventions is expected to increase as non-adherence has a marked effect on worsened economic outcomes.^8^

### Strengths and limitations

By using real-world and up-to-date data, the results of this study are expected to be representative for the current situation in the community. However, whereas relative results are expected to be generalisable between countries, absolute results may be less generalisable because of intensive monitoring and lung revalidation programmes currently running in the Netherlands, which may encourage more adherent behaviour. Some late onset asthma patients may be incorrectly classified as COPD patients as the database does not provide a diagnosis. In addition, some severe exacerbations (requiring hospital admission) may have been missed as the database only collects data from community pharmacies. Although we tried to use the dispensing of short courses of oral corticosteroids as a proxy for disease severity, findings could still be confounded by COPD severity, as neither lung function data nor a full set of proxies for COPD severity were available. The last two limitations are related to the size of the sample. It might be possible that the observed non-difference in persistence between the two inhaler types resulted from an insufficient power to detect the persistence difference. Another possibility is that the persistence difference might only exist, for example, in those COPD patients using their inhaler four times per day, and only a small proportion of the studied subjects used the inhaler at this high frequency.

### Implications for future research, policy and practice

In order to optimise patients’ persistence with COPD medication, we recommend a choice of inhaler depending on patients’ individual abilities to handle the inhaler.^33^ Besides, factors relating to optimal prescription, taking into account patients’ preferences^34^ and interventions on behaviour modification (for example, stimulation of self-management and inhalation techniques), are equally important and may add to patients’ capacity of acting in a more adherent behaviour.^35^ In addition, enhancing health-care providers’ education and communication skills may be beneficial.^36^ Targeting these interventions on non-adherent patients is expected to improve cost effectiveness.^8,37^

### Conclusions

The choice of an appropriate inhaler device is an important issue regarding COPD patients’ persistence; however, regarding the inhaler being a multi-dose or a single-dose device does not seem to affect patients’ persistence with LABAs. Over 80% of patients, who initially seemed to discontinue LABAs, re-started their initial medication or switched to a different type of inhaler or medication within 1 year.

## Figures and Tables

**Table 1:** Patient characteristics (*N*=575)

	*Multiple-dose inhalers (*N*=475)*		*Single-dose inhalers (*N*=100)*		
*Patient characteristics*	N	*%*	N	*%*	P-*value*
*Gender*					
Male	226	47.6	55	55.0	0.19^a^
					
*Age*					
Mean (s.d.)	69.3 (8.9)		69.6 (9.2)		0.75^b^
					
*Initial prescriber*					
Specialist	56	11.8	27	27.0	*<0.05* a
					
*Socioeconomic status*					
Mean (s.d.)	0.6 (1.1)		0.6 (1.1)		0.67^b^
					
*Comorbidity*					
Osteoporosis	21	4.4	2	2.0	0.40^a^
Diabetes mellitus	70	14.7	12	12.0	0.53^a^
Ischaemic heart disease	36	7.6	10	10.0	0.42^a^
Heart failure	73	15.4	21	21.0	0.18^a^
Disorders of lipid metabolism	134	28.2	16	16.0	*<0.05* a
Other cardiovascular disorders	271	57.1	52	52.0	0.38^a^
Depression	45	9.5	9	9.0	0.99^a^
Dementia	1	0.2	2	2.0	0.08^a^
Anxiety	123	25.9	26	26.0	0.99^a^
Psychosis	7	1.5	6	6.0	*<0.05* a
Rheumatic arthritis	76	16.0	13	13.0	0.54^a^
Thyroid disease	28	5.9	3	3.0	0.33^a^
					
*Number of comorbidities*					
Mean	1.9 (1.6)		1.7 (1.5)		0.42^b^
					
*Short-acting bronchodilators*					
At least one prescription	94	19.8	23	23.0	0.50^a^
					
*Short courses oral corticosteroids (yearly)*					
Mean	0.5 (2.1)		0.4 (0.7)		0.36^b^
					
*Initial medication*					
Formoterol	224	47.2	45	45.0	*<0.05* a
Indacaterol	0	0.0	9	9.0	
Salmeterol	251	52.8	46	46.0	
					
*Dosing regimen*					
Mean	2.00 (0.50)		1.89 (0.55)		*<0.05* b

Italic, significant (*P*<0.05).

a Two-sided Fisher’s exact test.

b Mann–Whitney *U*-test.

## Notes

TvdM has received research grants from AstraZeneca, GlaxoSmithKline, Nycomed, MSD, Almirall, consultancy fees for advisory boards from AstraZeneca, Nycomed, MSD, Novartis, Almirall and speaker fees from AstraZeneca, GlaxoSmithKline, Nycomed, Novartis and MSD. PNRD has received reimbursements for attending symposia, fees for speaking, organising educational events, funds for research or fees for consulting from AstraZeneca, Boehringer-Ingelheim, Chiesi, MSD, Mundipharma, Novartis, Takeda, Almirall and Teva. MJP has received grants, honoraria and travel stipends from various pharmaceutical companies including GlaxoSmithKline. The remaining authors declare no conflict of interest.
